# A New N6‐Methyladenosine Inhibitor, Celastrol, Alleviates Rheumatoid Arthritis via Targeting IGF2BP3

**DOI:** 10.1002/mco2.70431

**Published:** 2025-10-28

**Authors:** Qishun Geng, Yi Jiao, Wenya Diao, Jiahe Xu, Zhaoran Wang, Xing Wang, Zihan Wang, Lu Zhao, Lei Yang, Yilin Wang, Kan Wang, Tingting Deng, Bailiang Wang, Cheng Xiao

**Affiliations:** ^1^ Department of Pharmacy The First Affiliated Hospital of Zhengzhou University Zhengzhou Henan China; ^2^ China–Japan Friendship Clinical Medical College Chinese Academy of Medical Sciences & Peking Union Medical College Beijing China; ^3^ Institute of Clinical Medical Sciences China–Japan Friendship Hospital Beijing China; ^4^ Beijing University of Chinese Medicine China–Japan Friendship Hospital Clinical Medicine Beijing China; ^5^ Peking University China–Japan Friendship School of Clinical Medicine Beijing China; ^6^ Department of TCM Rheumatology China–Japan Friendship Hospital Beijing China; ^7^ China–Japan Friendship Hospital Capital Medical University Beijing China; ^8^ Department of Pathology China–Japan Friendship Hospital Beijing China; ^9^ Beijing Friendship Hospital Capital Medical University Beijing China; ^10^ Department of Anesthesiology China–Japan Friendship Hospital Beijing China; ^11^ Department of Orthopaedic Surgery China–Japan Friendship Hospital Beijing China; ^12^ Department of Emergency China–Japan Friendship Hospital Beijing China

**Keywords:** celastrol, cell proliferation, IGF2BP3, inflammatory activation, rheumatoid arthritis

## Abstract

The proliferation of fibroblast‐like synoviocytes (FLS) and macrophage‐mediated inflammation are the main clinical features of rheumatoid arthritis (RA). Studies showed that insulin‐like growth factor‐2 mRNA binding protein‐3 (IGF2BP3) may be involved in regulating the biological functions of different immune cells and FLS. Therefore, the identification of drugs that target IGF2BP3 has important clinical significance for improving RA. Molecular docking and surface plasmon resonance (SPR) analyses were used to identify a small molecule compound targeting IGF2BP3, celastrol (CEL). We subsequently examined the effects of CEL on RAW264.7 cells and FLS. IGF2BP3 knockout (KO) arthritis mice were used to identify the targets and mechanism of CEL in relieving RA. We found that CEL could bind to IGF2BP3 closely and reduce its expression. Additionally, CEL not only inhibited RA‐FLS proliferation but also decreased the inflammatory activation of macrophages. The IGF2BP3–RASGRF1–mTORC1 was critical for CEL‐mediated amelioration of RA. KO‐IGF2BP3 arthritis mice further showed that the protective effect of CEL against arthritis depended on IGF2BP3. Collectively, this study revealed that CEL inhibited the IGF2BP3/RASGRF1/mTORC1 axis to reduce cell proliferation and inflammatory activation, thereby alleviating the progression of RA. Our study suggests that clinical attention should be given to IGF2BP3 inhibitors, such as CEL.

## Graphical abstract

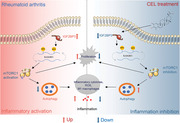

## 1 Introduction

Rheumatoid arthritis (RA) is an autoimmune disease whose causes are related to various factors such as genetics and environments [1]. Local synovial inflammatory response and progressive bone erosion are the main clinical manifestations of RA. The invasion of fibroblast‐like synoviocytes (FLS) and the activation of macrophage inflammation are the main causes of joint damage [2, 3]. RA‐FLS exhibit a proliferative and invasive phenotype, as well as autonomous pathogenic characteristics [4]. In addition, many synovial macrophages (SMs) are present in the RA synovial membrane; these cells not only are dominated by the M1 phenotype, but can also interact with FLS, monocytes, and osteoclasts to increase the secretion of proinflammatory cytokines, including tumor necrosis factor (TNF)‐α, interleukin (IL)‐1β, and IL‐6 [5]. At present, although targeted therapy and immunotherapy have significantly improved the prognosis of RA, the morbidity, disability, and mortality associated with RA are increasing annually, causing heavy burdens on patients and society [6]. Therefore, it is clinically important to determine the underlying mechanism by which FLS and macrophages affect the development of RA, discover new targets for treating RA, and identify effective drugs for RA treatment.

Previous studies have shown that both genetic and epigenetic factors can affect the occurrence of RA [7]. Among them, abnormalities in N6‐methyladenosine (m^6^A) modification are related to the progression of RA. At present, the role of m^6^A modification is unclear in RA [8, 9]. A number of studies have reported that the m^6^A modification plays a role in various immune cells, including B cells, macrophages, and T cells [10, 11]. And, many m^6^A modification‐related enzymes are involved in the regulation of biological processes in synovial cells and macrophages [12, 13, 14]. Our previous results revealed that insulin‐like growth factor‐2 mRNA binding protein‐3 (IGF2BP3) was closely related to synovial hyperplasia and macrophage M1 polarization. This finding suggests that IGF2BP3 can be a potential target for therapeutic intervention in RA [15, 16]. The search for small‐molecule compounds targeting IGF2BP3 in synovial cells or macrophages to inhibit cell proliferation and inflammatory activation may provide a promising treatment for RA. However, drug development based on m^6^A modifications is very rare. Although researchers have found the inhibitors of m^6^A‐modified demethylases (fat mass and obesity‐associated [FTO]), preclinical studies are still needed to confirm their effect. At present, there are no drugs targeting m^6^A‐modified enzymes for the treatment of RA clinically [17, 18].

*Tripterygium wilfordii* Hook. f. has the effects of dispelling wind, removing dampness, reducing swelling, relieving pain, activating channels, clearing heat, and detoxifying [19, 20]. Various studies have shown that *T. wilfordii* Hook. f. can reduce stiffness, joint swelling, and joint pain in RA patients, improve function, increase grip strength, and decrease the erythrocyte sedimentation rate (ESR) and the rheumatoid factor (RF) [21]. Modern pharmacological studies have also shown that *T. wilfordii* Hook. f. has strong immunomodulatory, anti‐inflammatory, and antitumor activities [22]. At present, *T. wilfordii* Hook. f. has been widely used in the treatment of RA, but its mechanism is unclear. Celastrol (CEL) is one of the most important components of *T. wilfordii* Hook. f., has strong antioxidant, anti‐cancer, angiogenesis, anti‐rheumatoid, and other effects. At present, many studies are available describing the mechanism by which CEL ameliorates RA [23]. CEL inhibits the reactive oxygen species (ROS)‐NF‐κB axis, which participates in the regulation of the NLRP3 inflammasome, thereby alleviating joint swelling and inflammatory cell infiltration in CIA rats [24]. CEL can also reduce synovial hyperplasia and the release of inflammatory cytokines, thereby alleviating the symptoms of arthritis in mice [25]. However, owing to its toxicity and side effects, the clinical application of CEL is severely limited [26]. Therefore, further exploration of the targets of CEL and the mechanism by which CEL alleviates RA is critical for promoting the modernization of traditional Chinese medicine.

Given that IGF2BP3 is critical for synovial hyperplasia and macrophage M1 polarization, we searched for small‐molecule compounds targeting IGF2BP3 by molecular docking and surface plasmon resonance (SPR) analyses. The results showed that CEL could bind to IGF2BP3 closely and reduce its expression. Moreover, CEL reduced the expression of IGF2P3 and RASGRF1 in the synovium of rats with collagen‐induced arthritis (CIA). IGF2BP3 knockout (KO) arthritis mice also showed that the arthritis protective effect of CEL depends on IGF2BP3. These data provide new and valuable insights into the molecular mechanisms of RA and suggest that IGF2BP3 inhibitors, such as CEL, require clinical attention.

## 2 Results

### 2.1 IGF2BP3 is the Direct Target of CEL

**FIGURE 1:**
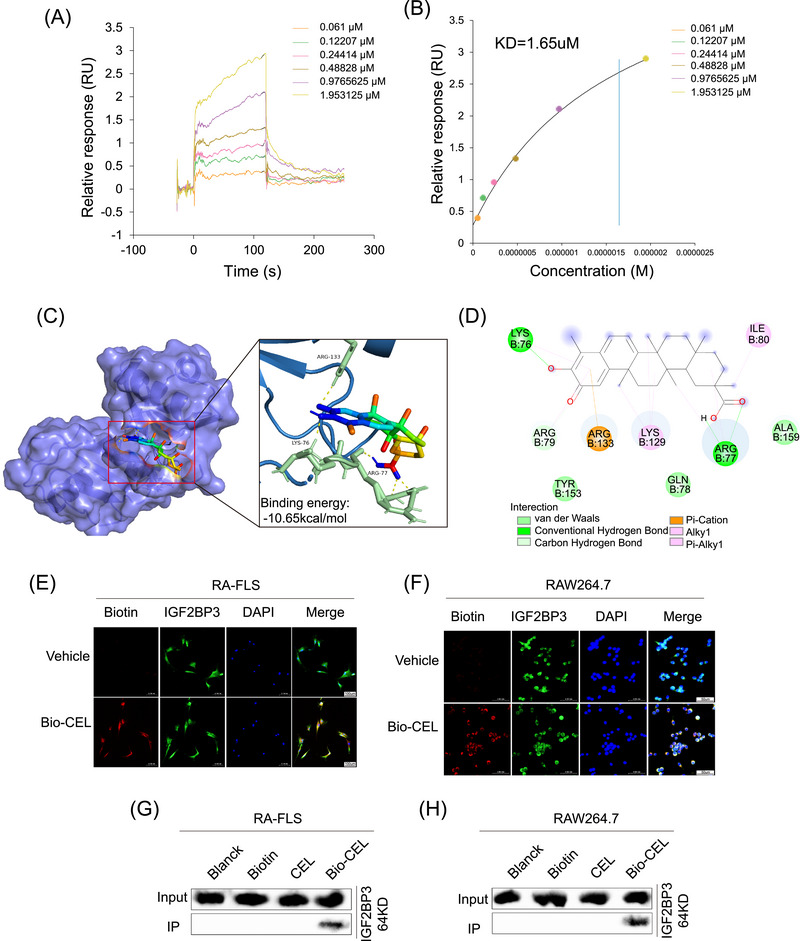
Discovery of CEL as an IGF2BP3‐targeting small molecule. (A and B) IGF2BP3 exhibited potent binding ability with CEL, as determined by SPR (KD = 1.65 µM). The 3D (C) and 2D (D) binding conformations between CEL and IGF2BP3 (6FQR). (E and F) The colocalization of Bio‐CEL with IGF2BP3 was detected by fluorescence microscopy. (G and H) Bio‐CEL interacted with IGF2BP3 in RA‐FLS and RAW264.7 cells. *p < 0.05, **p < 0.01, ***p < 0.001.

In a previous study, we analyzed the role of 19 m^6^A methylation regulators in RA and reported that IGF2BP3 not only has important diagnostic value for RA but also participates in the regulation of synovial hyperplasia and macrophage M1 polarization [15, 16]. Thus, the search for small‐molecule compounds targeting IGF2BP3 has clinical significance for RA treatment. SPR analysis was performed to explore the binding ability between IGF2BP3 and small molecule compounds that have therapeutic effects on RA, including celastrol, triptolide, medicarpin, curcumin, curbitacin B, and epigallocatechin (Figure S1A–E). Interestingly, there is a strong binding ability between CEL and IGF2BP3, with a dissociation constant (KD) of 1.65 µM (Figure 1A,B).

To further identify whether IGF2BP3 is the target of CEL, the binding score was evaluated through molecular docking, which showed the high binding affinities between CEL and IGF2BP3 (CEL–IGF2BP3 (PDB ID: 6FQR): −10.65 kcal/mol; CEL–IGF2BP3 (PDB ID: 6GQE): −7.82 kcal/mol). The binding poses and binding sites for CEL and IGF2BP3 are shown in Figure 1C and Figure S2A. Figure 1D shows that CEL is bound to IGF2BP3 (PDB ID: 6FQR) at ARG‐133, LYS‐76, and ARG‐77. Figure S2B shows that CEL is bound to IGF2BP3 (PDB ID: 6GQE) at VAL‐231 and SER‐227.

For a more complete and accurate understanding of the binding process, molecular dynamics (MD) simulations of CEL and IGF2BP3 were performed. The backbone root mean square deviation (RMSD) values of the CEL/IGF2BP3 (PDB ID: 6GQE) and CEL/IGF2BP3 (PDB ID: 6FQR) tended to converge, indicating whole system equilibrium (Figure S2C,D,H,I). The radius of gyration curve also showed that the structure of docked complexes tended to be stabilized (Figure S2E,J). The conformations were similar before and after the MD simulation (Figure S2F,G,K,L).

To confirm this celastrol–IGF2BP3 interaction in intact systems, we used biotin‐celastrol (Bio‐CEL). Immunofluorescent colocalization analysis demonstrated the direct binding of Bio‐CEL and IGF2BP3 (Figure 1E,F). Meanwhile, we performed pull‐down assays with lysates from RA‐FLS and RAW264.7 cells, whose results revealed that Bio‐CEL was bound to IGF2BP3 protein in lysates from both RA‐FLS and RAW264.7 cells (Figure 1G,H). In addition, proteomic analysis based on pull‐down assay of RA synovial tissue lysate also showed that Bio‐CEL binds to IGF2BP3 protein (Figure S2M).

Collectively, these findings suggest that CEL directly targeted IGF2BP3 and reduced the expression of IGF2BP3 in RA‐FLS and RAW264.7 cells.

### 2.2 CEL Can Reduce IGF2BP3 Expression, Inhibit Synovial Hyperplasia, and Macrophage M1 Polarization

**FIGURE 2:**
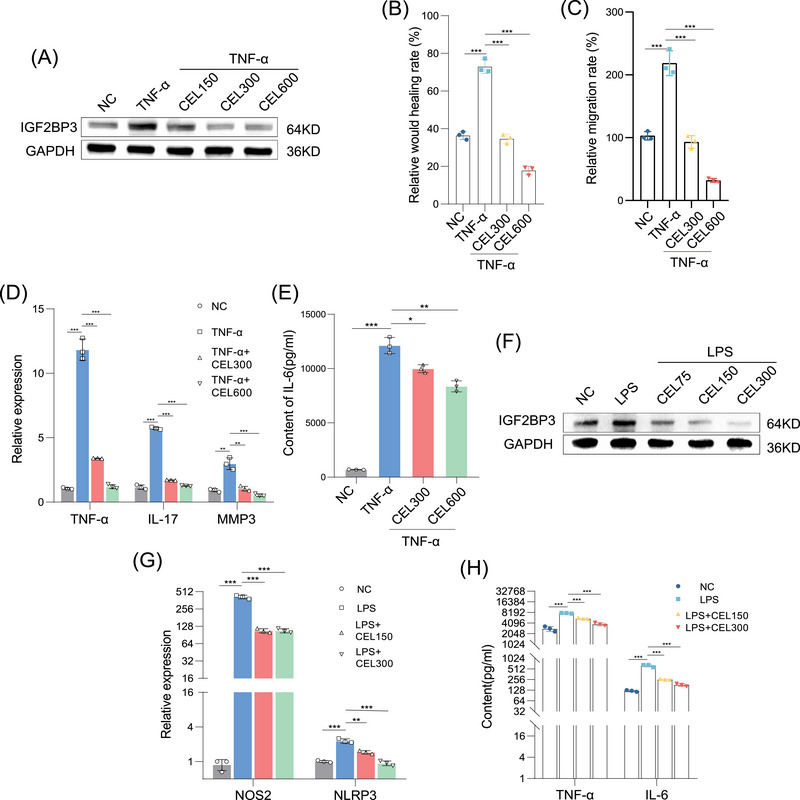
CEL inhibits RA‐FLS proliferation and M1 macrophage polarization. (A) The protein expression levels of IGF2BP3 in RA‐FLS after CEL treatment for 24 h. The quantification of scratch healing assays (B) and Transwell assays (C) of RA‐FLS treated with CEL for 24 h. (D) The effect on TNF‐α, IL‐17, and MMP3 expression in RA‐FLS treated with CEL for 24 h. (E) The content of IL‐6 secreted by RA‐FLS. (F) The protein expression levels of IGF2BP3 in RAW264.7 cells after treatment with CEL. (G) The mRNA expression level of NOS2 and NLRP3 in RAW264.7 cells. (H) The content of TNF‐α and IL‐6 secreted by RAW264.7 cells treated with CEL for 24 h. *p < 0.05, **p < 0.01, ***p < 0.001.

Given the ability of IGF2BP3 in inhibiting synovial hyperplasia and macrophage M1 polarization, we explored the role of CEL in RA‐FLS and macrophages. The CCK‐8 assay indicated that CEL was not cytotoxic to RA‐FLS in the range of 0–300 nM (Figure S3A). In addition, we found that CEL decreased the protein expression levels of IGF2BP3 in RA‐FLS (Figure 2A, Figure S3B). A dose of 300 or 600 nM was selected to investigate whether CEL could prevent or inhibit the proliferation and inflammation in RA‐FLS. To simulate the chronic inflammation and tissue destruction environment in RA, we applied 10 ng/mL TNF‐α to RA‐FLS [27]. CEL significantly suppressed the wound‐healing ability (Figure 2B, Figure S3C) and migration ability (Figure 2C, Figure S3D) of RA‐FLS. CEL also inhibited the polymerization of F‐actin (Figure S3E). TUNEL staining and annexin V‐FITC/PI staining showed that CEL promoted cell apoptosis (Figure S3F,G). CEL increased the proportion of G2/M‐phase cells, which may cause G2/M‐cell cycle arrest (Figure S3H). In addition, CEL greatly reduced the expression levels of TNF‐α, IL‐17, and MMP3 (Figure 2D), and CEL decreased IL‐6 secretion in FLS (Figure 2E). These data demonstrate that CEL played an important part in the proliferation, migration, invasion, and inflammatory cytokine release of RA‐FLS.

Next, we further explored whether CEL prevents or inhibits LPS‐induced inflammation in RAW264.7 cells. The results of the CCK‐8 assay revealed that 0–150 nM CEL was not significantly cytotoxic to RAW264.7 cells (Figure S3I). CEL also decreased the protein expression levels of IGF2BP3 in RAW264.7 cells (Figure 2F, Figure S3J). A dose of 150 or 300 nM was selected to examine the effect of CEL on LPS‐induced RAW264.7 cells. We further simulated the innate immune response and acute inflammatory environment in RA by applying 200 ng/mL LPS to RAW264.7 cells [28]. CEL reduced the mRNA expression levels of inflammatory markers, including NOS2 and NLRP3 (Figure 2G), and CEL decreased TNF‐α and IL‐6 secretion (Figure 2H). In addition, LPS increased the proportion of CD86^+^ M1 macrophages and the generation of ROS, while these effects were partially counteracted by CEL (Figure S3K,L). Researchers have found that the excessive production of ROS is closely related to the activation of NLRP3 inflammasome, the maturation and secretion of inflammatory factors, and the polarization of M1 macrophages [29, 30]. Together, these data demonstrate that CEL reduced the production of ROS, thereby decreasing macrophage inflammation.

These results suggest that CEL exhibited effects similar to those of siIGF2BP3 in inhibiting synovial cell proliferation and macrophage inflammation, which suggests that CEL may play a role of targeting IGF2BP3.

### 2.3 CEL Alleviates Arthritis Progression and Decreases IGF2BP3 Expression in CIA Rats

**FIGURE 3:**
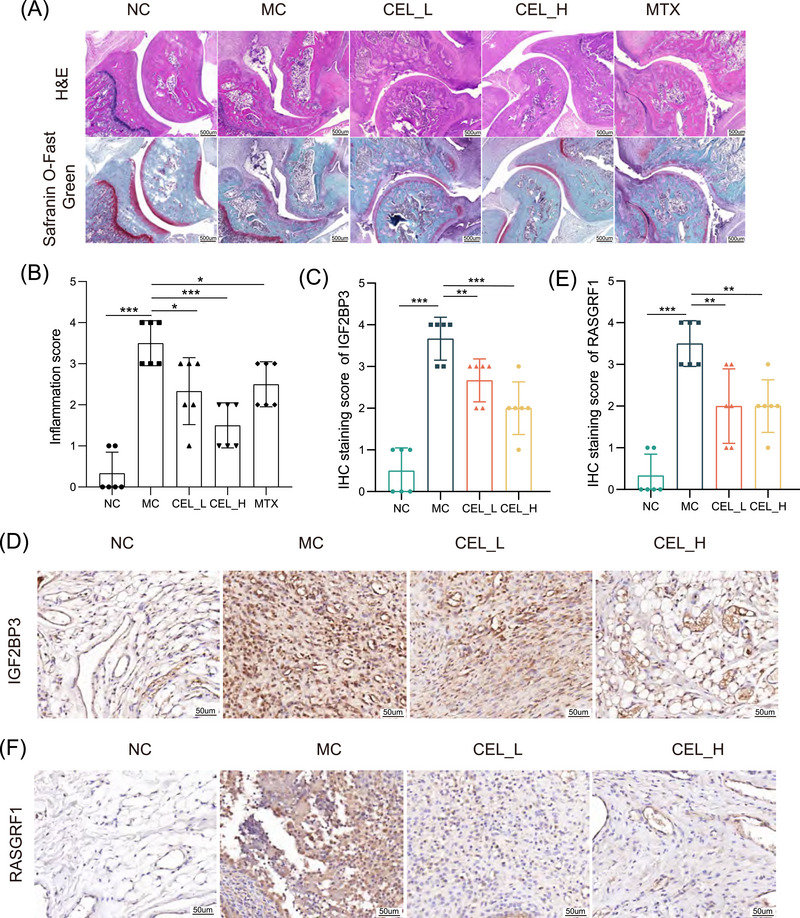
CEL alleviates bone damage and inflammatory infiltration, and inhibits the expression of IGF2BP3 in CIA rats. (A) Representative histology images showing H&E and safranin O/fast green staining are obtained from CIA rats after the indicated treatments. (B) Inflammation scores are measured. (C and D) Representative immunohistochemical assays and scores of IGF2BP3 in CIA rats’ synovial tissue. (E and F) Representative immunohistochemical assays and scores of RASGRF1 in CIA rats’ synovial tissue. *p < 0.05, **p < 0.01, ***p < 0.001.

The CIA rat model was used to assess the role of CEL in alleviating arthritis and inhibiting IGF2BP3 expression. The rats were given a high dose of CEL (1 mg/kg, CEL_H) or a low dose of CEL (0.5 mg/kg, CEL_L). The positive control group was given methotrexate daily by intragastric administration (Figure S4A). We found that CEL remarkably relieved arthritis score, paw thickness, and joint swelling (Figure S4B,C). In addition, CEL improved bone damage and inhibited synovial inflammatory infiltration and synovial cell proliferation, suggesting that CEL had a better therapeutic effect on CIA rats (Figure S4D,E, Figure 3A,B). Moreover, we found that CEL decreased the proportion of CD45^+^CD11b^+^CD86^+^ cells in the spleen (Figure S4F,G) and ameliorated the plasma contents of IL‐6 and TNF‐α (Figure S4H,I). These results demonstrate that CEL can alleviate RA progression in CIA rats. More importantly, the expression level of IGF2BP3 was robustly decreased in CEL‐treated rats, further suggesting that CEL could also alleviate RA progression by targeting IGF2BP3 in vivo (Figure 3C,D).

### 2.4 IGF2BP3 is a Direct Target of CEL to Inhibit Cell Proliferation and Promote Autophagy in RA‐FLS

Our previous study has found that RASGRF1‐mediated mTORC1 activation has a crucial role in IGF2BP3‐mediated promotion of cell proliferation and inhibition of autophagy in RA‐FLS [16]. Interestingly, CEL also inhibited the expression of RAGRF1 in CIA rats’ ankle, indicating that CEL participates in the regulation of IGF2BP3's downstream target (Figure 3E,F).

**FIGURE 4:**
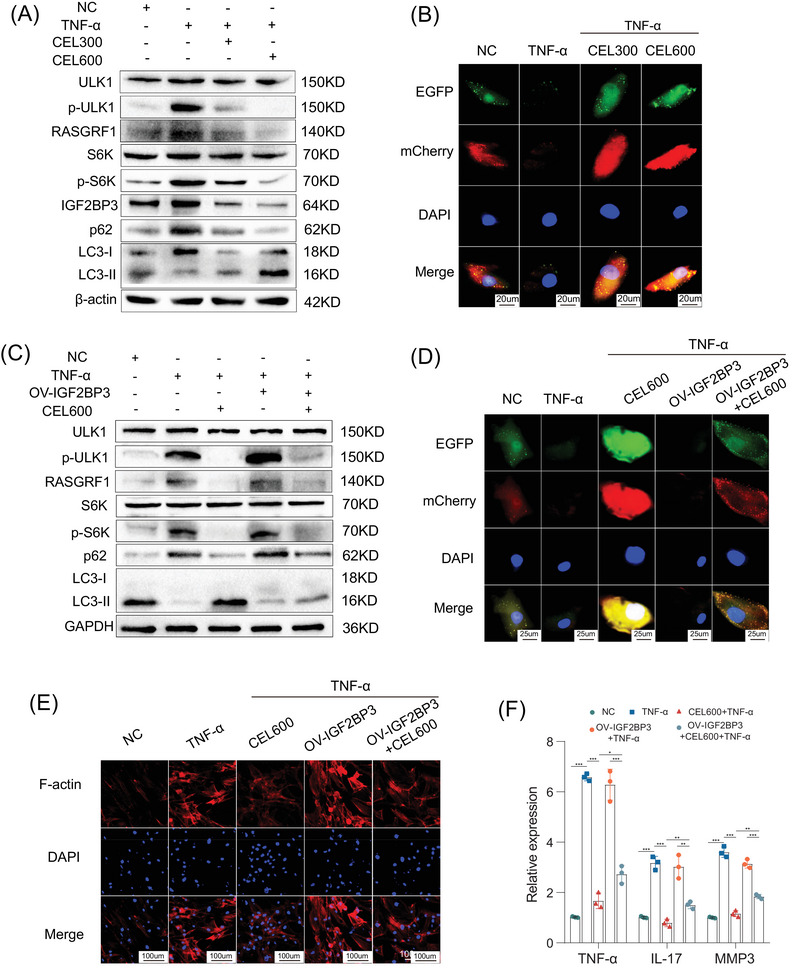
IGF2BP3 is a direct target of CEL to inhibit cell proliferation, inflammatory cytokine release, and promote autophagy in RA‐FLS. (A) Western blot analysis of the levels of ULK1, p‐ULK1, S6K, p‐S6K, RASGRF1, IGF2BP3, p62, and LC3 in RA‐FLS treated with TNF‐α or CEL. (B) Representative images of the RA‐FLS expressing mCherry‐GFP‐LC3. (C) Western blot analysis of the expression level of ULK1, p‐ULK1, S6K, p‐S6K, RASGRF1, IGF2BP3, p62, and LC3 in RA‐FLS after treatment with TNF‐α, CEL, or IGF2BP3 overexpression. (D) Representative images of RA‐FLS expressing mCherry‐GFP‐LC3 are shown after treatment with TNF‐α, CEL, or IGF2BP3 overexpression. (E) The expression of F‐actin in RA‐FLS. (F) The mRNA expression levels of TNF‐α, IL‐17, and MMP3 in RA‐FLS treated with TNF‐α, CEL, or OV‐IGF2BP3. *p < 0.05, **p < 0.01, ***p < 0.001.

To further determine the effect of CEL on the downstream target of IGF2BP3, we conducted in vitro experiments. Western blot analysis revealed that TNF‐α increased the expression of IGF2BP3 and RASGRF1, promoted the phosphorylation of ULK1 and S6K, and inhibited autophagy, whereas these effects were counteracted by CEL (Figure 4A). The markers of mCherry‐GFP‐LC3 also indicated that CEL promoted autophagy (Figure 4B). Immunofluorescence staining further revealed the colocalization of RASGRF1 (red) and IGF2BP3 (green), indicating a correlation between RASGRF1 and IGF2BP3 expressions. Moreover, TNF‐α increased the expression of IGF2BP3 and RASGRF1, which was reversed by CEL (Figure S5A). These results show that CEL regulates the downstream signaling of IGF2BP3.

To further determine whether IGF2BP3 is critical for CEL to inhibit cell proliferation and promote autophagy, we overexpressed IGF2BP3 and administered CEL stimulation in RA‐FLS. Western blot analysis revealed that CEL significantly decreased the expression of RASGRF1, inhibited the phosphorylation of ULK1 and S6K, and promoted autophagy; while these effects were counteracted by overexpression of IGF2BP3 (Figure 4C). Confocal microscopy also indicated that the autophagic flux was greatly promoted in the RA‐FLS stimulated with CEL, while the autophagic flux was partly decreased by overexpressing IGF2BP3 (Figure 4D). In addition, CEL significantly suppressed wound healing, migration, and invasion in FLS (Figure S5B–E). CEL inhibited the polymerization of F‐actin (Figure 4E). Annexin V‐FITC/PI staining and TUNEL staining also showed that CEL promoted cell apoptosis (Figure S5F,H). These effects were counteracted by the overexpression of IGF2BP3. The flow cytometry analysis in Figure S5G illustrated that the increased cell proportion in G2/M phase, stimulated by CEL, could be partially counteracted by overexpression of IGF2BP3. Also, IGF2BP3 reversed the decreased expression levels of TNF‐α, IL‐17, and MMP3, which were induced by CEL (Figure 4F). These results revealed that IGF2BP3 had a critical part in the ability of CEL to inhibit cell invasion and promote autophagy in RA‐FLS.

In addition, siRNA oligonucleotides targeting IGF2BP3 were transfected into RA‐FLS for validation. Loss of IGF2BP3 resulted in decreased IGF2BP3, RASGRF1, p62, p‐ULK1/ULK1, and p‐S6K/S6K levels in TNF‐α‐induced RA‐FLS. And, the addition of CEL did not yield a superior outcome (Figure S5I). Collectively, CEL inhibited cell proliferation, migration, invasion, and inflammatory cytokine release and promoted autophagy, at least in part by regulating IGF2BP3 in RA‐FLS.

### 2.5 CEL Inhibits IGF2BP3 to Attenuate M1 Macrophage Polarization and Inflammatory Activation Mediated by Autophagy

**FIGURE 5:**
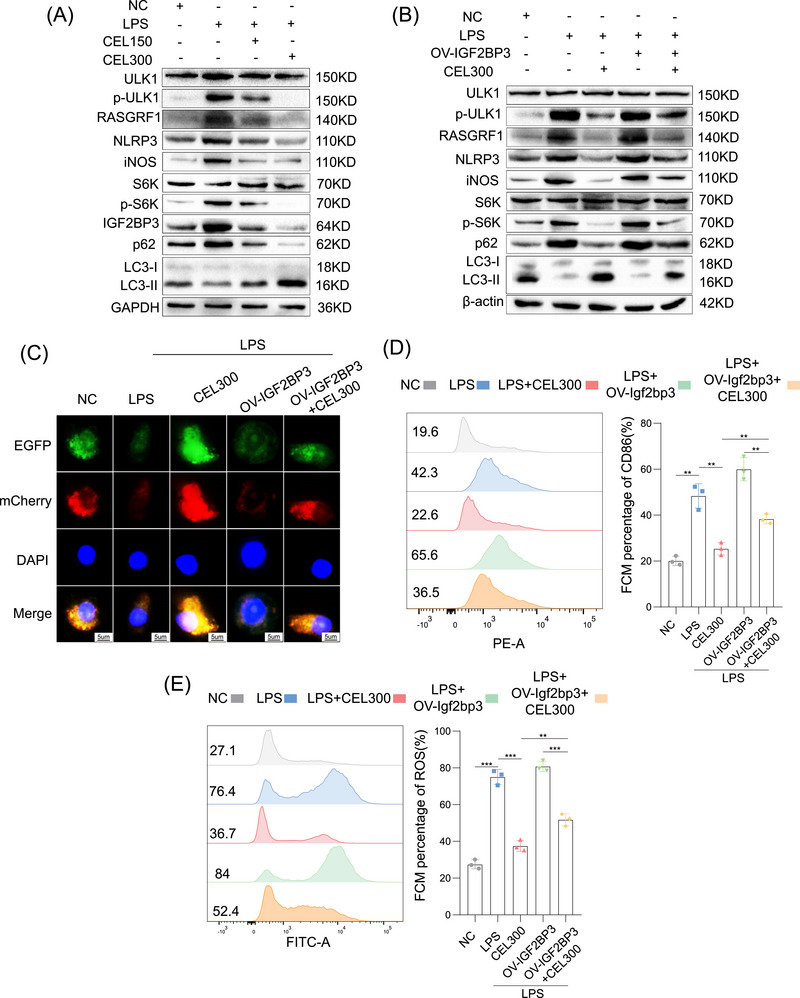
CEL inhibits IGF2BP3 to attenuate inflammatory activation in macrophages. (A) Western blot analysis of ULK1, p‐ULK1, S6K, p‐S6K, NLRP3, iNOS, RASGRF1, IGF2BP3, p62, and LC3 in RAW264.7 cells after treatment of LPS or CEL. (B) Western blot analysis of ULK1, p‐ULK1, S6K, p‐S6K, NLRP3, iNOS, RASGRF1, p62, and LC3 in RAW264.7 cells treated with LPS, CEL, or IGF2BP3 overexpression. (C) Representative images of RAW264.7 cells expressing mCherry‐GFP‐LC3 after treatment of LPS, CEL, or IGF2BP3 overexpression. The proportions of CD86^+^ cells (D) and ROS generation (E) in RAW264.7 cells treated with LPS, CEL, or IGF2BP3 overexpression. *p < 0.05, **p < 0.01, ***p < 0.001.

Next, we investigated the effects of CEL on IGF2BP3's downstream targets in macrophages. Given that studies have found IGF2BP3 promotes M1 macrophage polarization through RASGRF1‐mediated mTORC1 activation, we examined the effects of CEL on RASGRF1, mTORC1, and autophagy. Western blot analysis revealed that LPS increased the expression of IGF2BP3, RASGRF1, and inflammatory markers (NLRP3 and iNOS), promoted the phosphorylation of ULK1 and S6K, and inhibited autophagy in RAW264.7 cells; while these effects were counteracted by CEL (Figure 5A). There was also obvious colocalization of RASGRF1 (red) and IGF2BP3 (green), showing a correlation between RASGRF1 and IGF2BP3 expressions; and LPS increased the expression of IGF2BP3 and RASGRF1 in RAW264.7 cells, which was reversed by CEL (Figure S6A). In addition, confocal microscopy analysis indicated that CEL promoted autophagy (Figure S6B). TEM analysis showed that LPS decreased the number of autolysosomes, while CEL increased the number of autolysosomes in RAW264.7 cells (Figure S6C). In THP‐1 cells, LPS also greatly increased the expression of IGF2BP3, RASGRF1, and inflammatory markers (NLRP3 and iNOS), promoted the phosphorylation of ULK1 and S6K, and inhibited autophagy; but these effects were also counteracted by CEL (Figure S6D). Taken together, these results showed that CEL regulated downstream targets of IGF2BP3 in macrophages.

To further reveal that IGF2BP3 is critical for CEL‐mediated attenuation of M1 macrophage polarization, we overexpressed IGF2BP3 and administered CEL stimulation in RAW264.7 cells. CEL greatly decreased the expression of RASGRF1 and inflammatory markers (NLRP3 and iNOS), inhibited the phosphorylation of ULK1 and S6K, and promoted autophagy; whereas these effects were counteracted by the overexpression of IGF2BP3 (Figure 5B). Confocal microscopy showed that IGF2BP3 reversed the increase in autophagic flux stimulated by CEL (Figure 5C). The overexpression of IGF2BP3 also countered the decreased proportion of CD86^+^ M1 macrophages and ROS generation after treatment of CEL (Figure 5D,E). These data demonstrate that CEL promoted autophagy to decrease inflammatory activation, which was associated with IGF2BP3. In addition, siRNA oligonucleotides targeting IGF2BP3 were transfected into RAW264.7 cells for validation. Loss of IGF2BP3 resulted in decreased IGF2BP3, RASGRF1, NOS2 NLRP3, p62, p‐ULK1/ULK1, and p‐S6K/S6K levels in LPS‐induced RAW264.7 cells. However, the addition of CEL did not produce better results (Figure S6E). In summary, CEL decreased inflammatory activation, at least in part by regulating IGF2BP3 to promote autophagy in macrophages.

### 2.6 IGF2BP3‐Mediated mTORC1 Activation Plays a Critical Part in CEL‐Mediated Inhibition of Cell Proliferation and Inflammation

**FIGURE 6:**
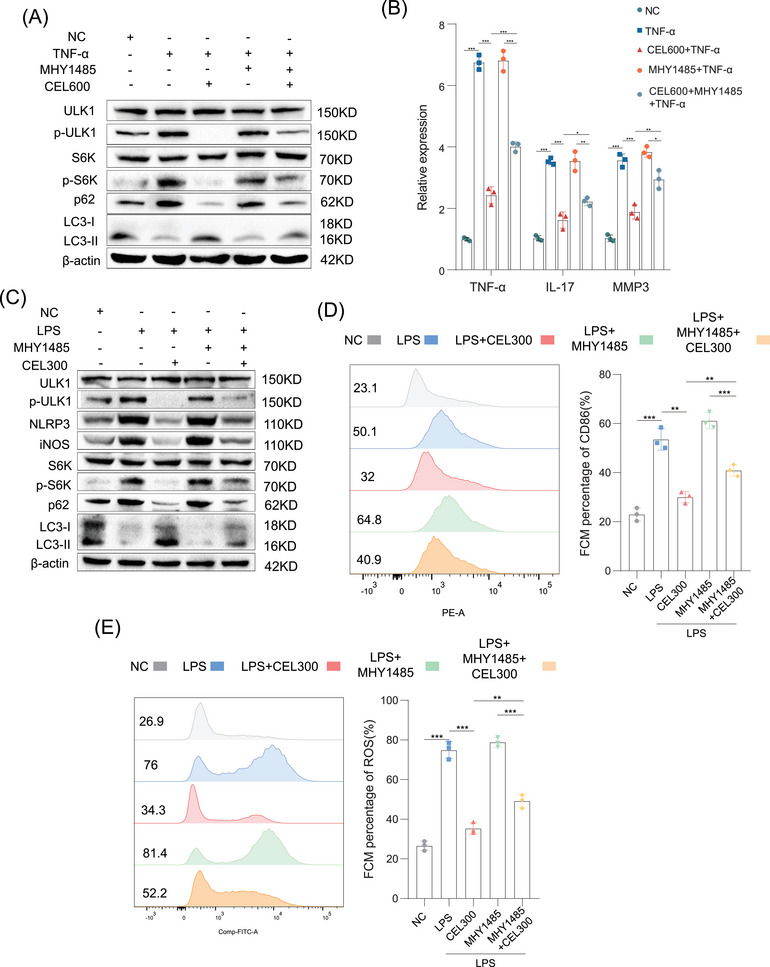
IGF2BP3‐mediated mTORC1 activation plays a significant role in CEL‐mediated inhibition of cell proliferation and inflammatory activation. (A) Western blot analysis of ULK1, p‐ULK1, S6K, p‐S6K, p62, and LC3 in RA‐FLS after treatment with TNF‐α, CEL, or MHY1485. (B) The mRNA expression levels of TNF‐α, IL‐17, and MMP3 in RA‐FLS after treatment with TNF‐α, CEL, or MHY1485. (C) Western blot analysis of ULK1, p‐ULK1, S6K, p‐S6K, NLRP3, iNOS, p62, and LC3 in RAW264.7 cells treated with LPS, CEL, or MHY1485. The proportions of CD86^+^ cells (D) and ROS generation (E) in RAW264.7 cells after treatment with LPS, CEL, or MHY1485. *p < 0.05, **p < 0.01, ***p < 0.001.

Researchers have found that mTORC1 promotes the phosphorylation of ULK1 to inhibit autophagy [31]. In addition, mTORC1 regulates cell growth and cell proliferation by mediating S6K phosphorylation to regulate protein synthesis and ribosome biogenesis [32]. To explore the role of IGF2BP3‐mediated mTORC1 activation in CEL‐mediated regulation of cell proliferation and inflammatory activation, we administered CEL and the mTORC1 activator, MHY1485, in RA‐FLS. MHY1485 antagonized CEL‐mediated inhibition of mTORC1 in RA‐FLS (Figure 6A), which indicated that CEL promoted autophagy by inhibiting mTORC1 activation. In addition, CEL reduced the expression levels of TNF‐α, IL‐17, and MMP3, which were partly counteracted by MHY1485 (Figure 6B). The flow cytometry results shown in Figure S6F reveal that the apoptosis of RA‐FLS increased after exposure to CEL. However, the increased proportion of apoptotic cells was partially counteracted by MHY1485. Collectively, these results revealed that IGF2BP3‐mediated mTORC1 activation plays a significant role in CEL‐mediated inhibition of cell proliferation and inflammatory cytokine release, and the promotion of autophagy in RA‐FLS.

Next, to further determine whether mTORC1 activation is critical for CEL to attenuate M1 macrophage polarization, RAW264.7 cells were administered CEL or MHY1485. Western blot analysis indicated that CEL decreased the expression of inflammatory markers (NLRP3 and iNOS), inhibited the phosphorylation of ULK1 and S6K, and promoted autophagy; while these effects were also counteracted by MHY1485 (Figure 6C). More interestingly, MHY1485 significantly abrogated the decreased proportion of M1 macrophages and ROS after the treatment of CEL (Figure 6D,E). In short, CEL activated autophagy to relieve macrophage inflammation by inhibiting IGF2BP3‐mediated mTORC1 activation.

### 2.7 IGF2BP3 is the Direct Target Through Which CEL Alleviates RA Progression

**FIGURE 7:**
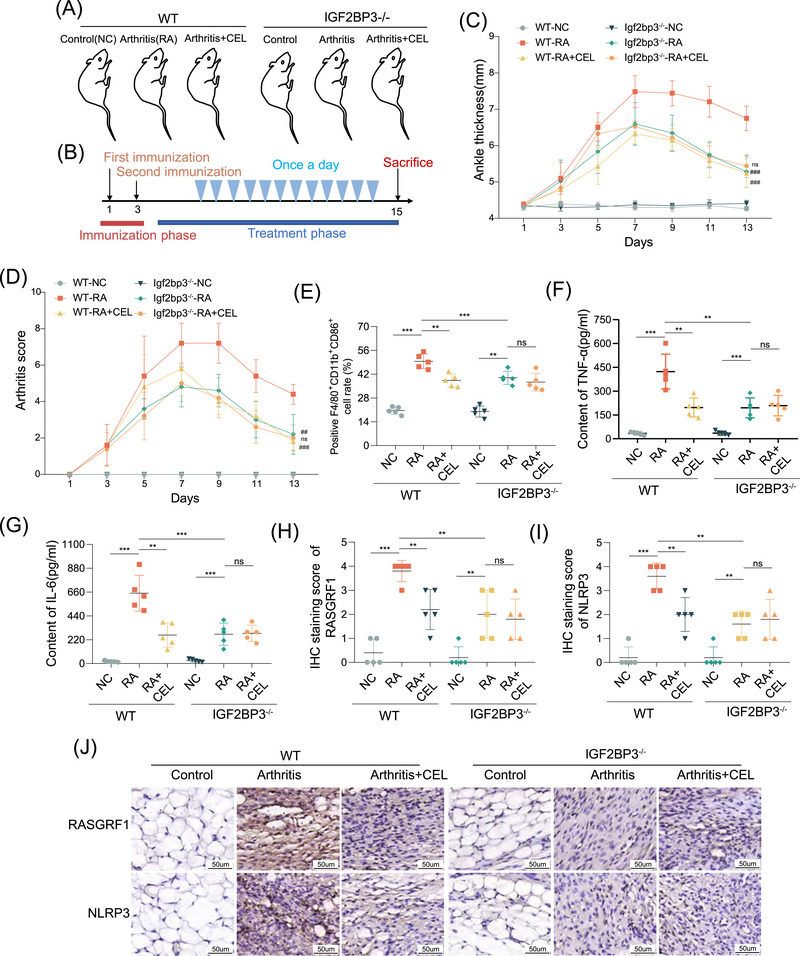
The knockout of IGF2BP3 abolishes the effects of CEL in alleviating the progression of RA. (A) The groups of animal experiments. (B) An illustration of the induction and treatment of mice with arthritis. (C) Hind paw thickness is detected from the second immunization. Compared with WT‐RA, #*p* < 0.05, ##*p* < 0.01, ###*p* < 0.001; compared with Igf2bp3^−/−^‐RA, ^ns^ *p* > 0.05, *p < 0.05, **p < 0.01, ***p < 0.001. (D) Arthritis scores are monitored from the second immunization. (E) The proportion of F4/80^+^CD11b^+^CD86^+^ M1 macrophages in the spleens of mice. The content of TNF‐α (F) and IL‐6 (G) in the serum of mice. (H–J) Representative immunohistochemical assays and scores of RASGRF1 and NLRP3 in the synovial tissue. ^ns^ *p* > 0.05, *p < 0.05, **p < 0.01, ***p < 0.001.

To determine whether IGF2BP3 is the primary target of CEL alleviating RA, we constructed an arthritis model in IGF2BP3 knockout (KO) mice. The grouping and treatment diagram are shown in Figure 7A,B. Genetic deletion of IGF2BP3 attenuated the arthritis score and ankle thickness in arthritic mice. Notably, the IGF2BP3 KO plus CEL combination did not show further protective effects (Figure 7C,D). Meanwhile, the arthrosis appearance, H&E, micro‐CT, and safranine O and green staining also revealed the improvements in synovial inflammation and cartilage destruction in the IGF2BP3 KO arthritis mice compared with the WT arthritis mice (Figure S7A–C); while CEL did not show further relieved effects in IGF2BP3 KO arthritis mice. Moreover, the IGF2BP3 KO plus CEL group did not show further inhibition effects on the proportion of F4/80^+^CD11b^+^CD86^+^ M1 macrophage, compared with that in the IGF2BP3 KO arthritis mice (Figure S7D, Figure 7E). In WT arthritis mice, the serum contents of TNF‐α and IL‐6 decreased with CEL treatment; the IGF2BP3 KO group and the IGF2BP3 KO plus CEL group presented the same trend (Figure 7F,G). In IGF2BP3 KO mice, the expression of IGF2BP3 was greatly decreased, which verified the knockout efficiency. And, CEL inhibited IGF2BP3 expression in WT arthritis mice (Figure S7E). In addition, the protein expression levels of RASGRF1 and NLRP3 were reduced by CEL in WT arthritis mice (Figure 7H–J). Further effects were not observed in IGF2BP3 KO arthritic mice treated with CEL. These results reveal that the arthritis protective effect of CEL depends on IGF2BP3.

## 3 Discussion

FLS mainly reside in the synovial membranes of joints [2]. Under inflammatory conditions, RA‐FLS exhibit characteristics similar to those of tumors, such as over‐proliferation, migration, and invasion, and can upregulate the expression of stroma‐degrading enzymes in joints to promote bone and cartilage destruction [33]. In addition, FLS interacts with macrophages to promote the formation of an inflammatory environment [34]. The infiltration of excessive macrophages in synovial tissue is regarded as an early sign of active RA [35]. Numerous clinical trials have shown [36] that the ratio of M1/M2 macrophages is unbalanced in RA patients. Macrophages can activate various immune cells by secreting many proinflammatory cytokines and chemokines, triggering an inflammatory cascade response that ultimately leads to cartilage degradation and bone destruction [3]. The regulation of M1/M2 macrophage homeostasis is beneficial for promoting RA inflammation resolution and tissue repair. Taken together, the inhibition of synovial hyperplasia and macrophage M1 polarization is important for RA remission. In the study, CEL not only played a major part in RA‐FLS for cell proliferation and inflammatory cytokine release but also reduced the production of ROS, thereby decreasing inflammatory activation in macrophages. These results further indicate the potential of CEL to treat RA.

Research has reported the abnormal m^6^A levels in RA patients’ PBMCs, whose role in the pathogenesis of RA remains unclear [37]. In recent years, several studies have reported abnormal m^6^A modifications in autoimmune diseases, including RA [8, 38]. Notably, METTL3 has been reported to promote RA‐FLS activation and the inflammatory response by activating the NF‐κB signaling pathway [39]. ALKBH5 levels in the RA patients’ synovial tissue were higher than those in the healthy control group. Moreover, the severity of arthritis in rats with CIA injected with ALKBH5‐shRNA was improved [12]. To further explore the significance of the m^6^A modification in RA, we constructed an RA diagnostic model to evaluate its importance in RA, indicating that IGF2BP3 was the most important factor [15]. IGF2BP3 is highly expressed in the synovium of RA patients and CIA rats. In addition, we found that IGF2BP3 regulated the RAS signaling pathway through post‐transcriptional modifications to activate the mTORC1 signaling pathway, thereby promoting synovial cell proliferation and macrophage inflammatory activation [16]. Based on the importance of IGF2BP3 in RA, we searched for moleculars targeting of IGF2BP3 by molecular docking and SPR analyses, which showed that CEL could bind to IGF2BP3 closely and reduce its expression. By overexpressing IGF2BP3 and using an mTORC1 activator (MHY1485), we further demonstrated that IGF2BP3 is a direct target through which CEL ameliorated RA progression. Moreover, CEL decreased the expression of IGF2BP3 and RASGRF1 in the synovial membranes of rats with CIA. And IGF2BP3 KO arthritis mice also revealed that the arthritis therapeutic effect of CEL depends on IGF2BP3.

Previous studies have shown that CEL has antioxidant, anti‐cancer, anti‐neovascularization, anti‐rheumatoid, and other effects [40]. However, due to the multi‐target characteristics of TCM monomers, the mechanism by which CEL regulates FLS and immune cells is still unclear. In this study, we found that CEL has the function of targeted inhibition of IGF2BP3. In addition, CEL reduced the expression level of RASGRF1 by inhibiting IGF2BP3, thereby inhibiting the activation of the RAS pathway. The Ras pathway participates in cell proliferation, migration, survival, differentiation, and fibrosis. Ras proteins also induce FLS activation and have a part in the immunopathogenesis of RA [41]. It has been reported that H‐Ras has increased levels in RA‐FLS, which is due to rasgrp1 overexpression. Studies have also shown that RasGRP1 is involved in the generation of MMP3 and the transformation phenotype of FLS [42]. In addition, TNF‐α activates macrophages via the Ras/MAPK/ERK pathway, which promotes M1‐type polarization [43]. The study indicated that IGF2BP3–RASGRF1‐mediated mTORC1 activation played a major role in the CEL‐mediated inhibition of cell proliferation and inflammatory cytokine release in RA‐FLS. Moreover, CEL activated autophagy to reduce inflammation by inhibiting IGF2BP3–RASGRF1‐mediated mTORC1 activation in macrophages. When IGF2BP3 was overexpressed or mTORC1 inhibitors were used, the anti‐proliferative and anti‐inflammatory effects of CEL were significantly reduced. Molecular docking revealed that CEL could inhibit IGF2BP3 expression by binding to the VAL‐231, SER‐227, ARG113, LYS‐76, and ARG‐77 sites of the IGF2BP3 protein, which needs further verification. Compared with previous studies, this study was the first to identify the target of CEL from the perspective of RNA methylation. The results also revealed the regulatory mechanism by which CEL affects RA‐FLS and macrophages, and confirmed the regulatory significance of the IGF2BP3/RASGRF1/mTORC1 axis in CEL‐mediated reductions in cell proliferation and inflammatory activation. This discovery provides new targets and strategies for identifying the molecular mechanism of RA, and provides important theoretical support for the clinical application of IGF2BP3 inhibitors. However, further studies and validation are needed to confirm the clinical application prospects.

In conclusion, this discovery revealed that CEL inhibited the IGF2BP3/RASGRF1/mTORC1 axis to reduce cell proliferation and inflammatory activation, thereby alleviating the progression of RA. These findings have important clinical significance and provide a new mechanism of CEL for the treatment of RA.

## 4 Materials and Methods

### 4.1 Cell Culture and Transfection

We extracted FLS from the articular synovial tissues of RA patients as previously described [44]. RAW264.7 and THP‐1 cells were obtained from the Chinese Academy of Sciences (Shanghai, China). FLS between Passages 4 and 10 were used for the experiments.

IGF2BP3 siRNA (siIGF2BP3), RASGRF1 siRNA (siRASGRF1), and control siRNA (siNC) were chemically synthesized by GenePharma Co. Ltd (Shanghai, China). The coding sequences of IGF2BP3 and RASGRF1 were subcloned and inserted into the PCDH‐CMV lentiviral vector (Tsingke Biotechnology Co. Ltd.). The mCherry‐GFP‐LC3 adenovirus was provided by Hanheng Co. Ltd. The siIGF2BP3 target sequences used were as follows: human si‐IGF2BP3: 5ʹ‐GCAAAGGATT CGGAAACTT‐3ʹ; mouse siIgf2bp3: 5ʹ‐GGAGGUGCUGGAUAGUUUACU‐3ʹ. The siIGF2BP3 target sequences used were as follows: human siIGF2BP3: 5ʹ‐CUAAAGCUUUGAUUGAUAATT‐3ʹ; mouse siIgf2bp3: 5ʹ‐GCAAAGGCTACCTGAGCAA‐3ʹ. The sequence of the shRNA targeting human IGF2BP3 was shown as 5ʹ‐GCTGCACTTCAGACGAATTAT‐3ʹ. Lipofectamine 3000 transfection reagent was used (Thermo Fisher Scientific Co. Ltd., USA).

### 4.2 Animal Experiments

Forty male Sprague–Dawley (SD) rats (6–8 weeks, 180–200 g) were divided into five groups: normal control, model control, CEL‐low dose, CEL‐high dose, and positive control. Except for those in the normal control group, the rats in the other groups were used to establish the CIA model [45]. According to previous experiments and the literature [24, 46], 0.5 mg/kg and 1 mg/kg CEL (34157‐83‐0, MACKLIN, China) were administered to the rats by intraperitoneal (i.p.) injection, respectively; the rats on the positive control group received 0.5 mg/kg methotrexate (MTX) every 3 days (133073‐73‐1, MACKLIN, China) by i.g. Beginning on Day 8, the rats were treated once per day for a total of 21 days. According to the previous standards, the paw thickness and the arthritis scores were assessed to evaluate the severity of arthritis [47]. After 21 days, the rats were sacrificed and analyzed.

IGF2BP3^−/−^ mice were purchased from Cyagen Biosciences. According to previous studies, serum from 8–9 weeks arthritis K/BxN mice was used to construct an arthritis mouse model [48]. After transfer of serum twice, on the first and third day, 1.5 mg/kg CEL was administered to the mice by i.p. injection once a day [49]. After 12 days of treatment, the mice were euthanized, and the histopathological changes of their ankles were analyzed.

### 4.3 Real‐Time Quantitative PCR

A FastPure Cell/Tissue Total RNA Isolation Kit V2 (RC112‐01, Vazyme, China) was used to extract mRNA. GAPDH and β‐actin were used as internal controls. Table S1 lists the primers used in this study. Specimen preparation and statistical evaluation were conducted in accordance with methods outlined in a prior study [50].

### 4.4 Western Blotting

Specimen preparation and statistical evaluation were conducted in accordance with methods outlined in a prior study [16, 50]. The bands were detected by an ECL Western blotting detection system, and quantitatively analyzed by ImageJ software.

### 4.5 Enzyme‐Linked Immunosorbent Assay

Serum and cell supernatants were collected. The samples were processed according to the protocol of the enzyme‐linked immunosorbent assay (ELISA) Kit (R&D Systems), and the contents of TNF‐α and IL‐6 were measured. Data analysis was conducted in accordance with methods outlined in a prior study [15].

### 4.6 Flow Cytometry

Anti‐CD86 (BioLegend, USA), anti‐F4/80 (BioLegend, USA), anti‐CD45 (BioLegend, USA), and anti‐CD11b (BioLegend, USA) antibodies were used to evaluate the proportions of M1 macrophages. The levels of ROS were determined by a ROS detection kit (S0033S, Beyotime, China). Specimen preparation and statistical evaluation were conducted in accordance with our prior study [15].

### 4.7 Immunohistochemistry

The synovial tissues of RA patients, OA patients, and healthy controls were provided by China–Japan Friendship Hospital. Specimen preparation and statistical evaluation were conducted in accordance with a prior study [15].

### 4.8 Surface Plasmon Resonance

The recombinant IGF2BP3 protein was covalently conjugated to a CM5 sensor chip surface through amine coupling chemistry following manufacturer‐recommended procedures. Prior to immobilization, the target molecule was resuspended in sodium acetate buffer (pH 4.5) to optimize electrostatic interactions. Serial dilutions of celastrol (CEL) served as mobile‐phase analytes in subsequent binding assays. Real‐time interaction profiles were processed with Biacore T200 Evaluation Software (version 2.0), with equilibrium binding analysis conducted through steady‐state fitting algorithms to quantify the molecular affinity constants [45].

### 4.9 Molecular Dynamics Simulations

Molecular dynamics (MD) simulations using the CHARMM36 force field were conducted using the GROMACS 5.0 software suite [51, 52]. The CHARMM General Force Field was employed to generate ligand topology parameters. The system was hydrated using the TIP3P water model within a dodecahedral periodic boundary box and neutralized with appropriate counterions. Energy minimization was achieved through the steepest descent optimization method. The energy, electrostatic, and van der Waals interactions were calculated via the particle‐mesh Ewald (PME) algorithm. The systems underwent sequential equilibration: first, a 20,000‐step equilibration in the canonical (NVT) ensemble, followed by an additional 20,000‐step equilibration under isothermal‐isobaric (NPT) conditions. Finally, a production MD run spanning 100 ns was executed with periodic boundary constraints, with a 2.0‐fs integration timestep [53, 54].

### 4.10 Statistical analysis

Statistical computations and data visualization were performed with R version 4.0.4 software (Institute for Statistics and Mathematics, Vienna, Austria; https://www.r‐project.org) and GraphPad Prism software (GraphPad Software, San Diego, CA, USA). All experimental procedures were conducted with three independent biological replicates. Intergroup comparisons were assessed with two‐tailed Student's *t*‐tests. Quantitative results are reported as mean ± standard deviation (SD), with a statistical significance threshold defined as *p* < 0.05.

## Author Contributions

Cheng Xiao, Bailiang Wang, Tingting Deng, and Kan Wang conceived the project and designed the study. Qishun Geng conducted the assays and acquired and analyzed the data. Wenya Diao and Yi Jiao helped with animal housing. Jiahe Xu, Zhaoran Wang, Xing Wang, Zihan Wang, Lu Zhao, Lei Yang, and Yilin Wang participated in some experiments and collected human samples. All authors have read and approved the final manuscript.

## Ethics Statement

Ethical approval of this study was granted by the ethical committee of China–Japan Friendship Hospital with the ethical approval numbers 2024‐KY‐019 and 2024‐KY‐227. The ethical committee approved the exemption of informed consent from all participants. All animal experimental procedures were approved by the Ethics Committee of the Institute of Clinical Medical Sciences of China–Japan Friendship Hospital (ZRDWLL230074).

## Conflicts of Interest

The authors declare no conflicts of interest.

## Supporting information




**Figure S1**. SPR is used to determine the binding ability between IGF2BP3 and natural compounds, including triptolide (A), medicarpin (B), curcumin (C), curbitacin B (D), and epigallocatechin (E).
**Figure S2**. The auto‐docking, molecular dynamics (MD), and proteomic analysis of CEL and IGF2BP3. The 3D (A) and 2D (B) binding conformations between CEL and IGF2BP3 (6GQE). The RMSD curve of the MD process of the CEL/IGF2BP3 complexes (PDB ID: 6GQE) (C: proteins; D: CEL). (E) The radius of gyration curves of the MD process of docked complexes (CEL/IGF2BP3(PDB ID: 6GQE). The comparison of conformations before and after MD simulations of the CEL/IGF2BP3 complexes (PDB ID: 6GQE) (F: 0 ns; G: 100 ns). The RMSD curve of the MD process of the CEL/IGF2BP3 complexes (PDB ID: 6FQR) (H: proteins; I: CEL). (J) The radius of gyration curves of the MD process of the docked complexes CEL/IGF2BP3 (PDB ID: 6FQR). The comparison of conformation before and after MD simulations of the CEL/IGF2BP3 complexes (PDB ID: 6FQR) (K: 0 ns; L: 100 ns). (M) Mass spectrum peak of the IGF2BP3 protein.
**Figure S3**. CEL inhibits RA‐FLS proliferation and M1 macrophage polarization. (A) The viability of RA‐FLS was analyzed by the CCK8 assay. (B) The protein expression levels of IGF2BP3 in RA‐FLS after treatment with CEL for 24 h. Representative images of scratch assays (C) and Transwell assays (D) of RA‐FLS treated with CEL for 24 h. (E) The effect of CEL on F‐actin expression in RA‐FLS. (F) TUNEL (green) staining of RA‐FLS. (G) RA‐FLS apoptosis is measured with an annexin V‐FITC/PI staining assay after CEL treatment for 24 h. (H) Flow cytometric analysis is used to evaluate the cell cycle distribution of RA‐FLS. (I) The CCK8 assay is used to examine the viability of RAW264.7 after CEL treatment for 24 h. (J) The protein expression levels of IGF2BP3 in RAW264.7 cells after CEL treatment for 24 h. The proportion of CD86^+^ cells (K) and ROS content (L) in RAW264.7 cells treated with control, LPS, or CEL. *p < 0.05, **p < 0.01, ***p < 0.001.
**Figure S4**. CEL alleviates arthritis progression in CIA rats. (A) An illustration of the induction and treatment of rats with CIA. (B) Arthritis scores are monitored once every 3 days. (C) Hind paw thickness is calculated after the second immunization. Compared with the NC group, #*p* < 0.05, ##*p* < 0.01, ###*p* < 0.001. Compared with the MC group, *p < 0.05, **p < 0.01, ***p < 0.001. (D) Paw photographs of rats on 22nd day and the micro‐CT analysis of paws. (E) The BS/BV in the ankle is examined by micro‐CT. (F and G) The proportions of CD45^+^CD11b^+^CD86^+^ cells in the spleens of rats. The contents of TNF‐α(H) and IL‐6 (I) in the serum of rats. *p < 0.05, **p < 0.01, ***p < 0.001.
**Figure S5**. IGF2BP3 is a direct target of CEL to inhibit cell proliferation and inflammatory activation. (A) The immunofluorescence staining of IGF2BP3 and RASGRF1 in RA‐FLS after treatment with TNF‐α or CEL. The quantification of scratch healing assay (B and D) and Transwell assay (C and E) in RA‐FLS after treatment with TNF‐α, CEL, or IGF2BP3 overexpression. (F) The percentage of apoptotic RA‐FLS is measured with an annexin V‐FITC/PI staining assay. (G) Flow cytometric analysis is used to evaluate the cell cycle distribution of RA‐FLS. (H) Tunnel (green) staining in RA‐FLS. (I) Western blot analysis of the levels of ULK1, p‐ULK1, S6K, p‐S6K, RASGRF1, IGF2BP3, p62, and LC3 in RA‐FLS after treatment with TNF‐α, CEL, or siIGF2BP3. *p < 0.05, **p < 0.01, ***p < 0.001.
**Figure S6**. IGF2BP3‐mediated mTORC1 activation plays a significant role in CEL‐mediated inhibition of cell proliferation and inflammatory activation. (A) The immunofluorescence staining of IGF2BP3 and RASGRF1 in RAW264.7 cells treated with CEL or LPS for 24 h. (B) Representative images of RAW264.7 cells expressing mCherry‐GFP‐LC3. (C) TEM analysis of RAW264.7 cells treated with LPS or CEL. (D) Western blot analysis of ULK1, p‐ULK1, S6K, p‐S6K, NLRP3, iNOS, RASGRF1, IGF2BP3, p62, and LC3 in THP‐1 cells after treatment of LPS or CEL. (E) Western blot analysis of ULK1, p‐ULK1, S6K, p‐S6K, iNOS, NLRP3, RASGRF1, IGF2BP3, p62, and LC3 in RAW264.7 cells after treatment with LPS, CEL, or siIGF2BP3. (F) Flow cytometric analysis is used to evaluate the apoptosis proportion of RA‐FLS treated with control, LPS, CEL, or MHY1485. *p < 0.05, **p < 0.01, ***p < 0.001.
**Figure S7**. The knockout of IGF2BP3 abolishes the effects of CEL in alleviating the progression of RA. (A) Representative histology images of H&E, safranin O/fast green staining, arthrosis appearance, and micro‐CT are obtained from the mouse's ankle. (B) The BS/BV in the ankle is measured by micro‐CT. (C) The H&E scores are assessed. (D) The proportion of F4/80^+^CD11b^+^CD86^+^M1 macrophages in the spleens of mice. (E) Representative immunohistochemical assays and scores of IGF2BP3 in mice's synovial tissue. *p < 0.05, **p < 0.01, ***p < 0.001.
**Table S1**. The sequences of the primers used for RT‐qPCR.

## Data Availability

The authors have nothing to report.
