# Supplementary material for: A New N6‐Methyladenosine Inhibitor, Celastrol, Alleviates Rheumatoid Arthritis via Targeting IGF2BP3
Source: MedComm (2020). 2025 Oct 28;6(11):e70431. doi: 10.1002/mco2.70431 (PMC12559910; doi:10.1002/mco2.70431)
Supplement: Supplementary file 1 — Figure S1. SPR is used to determine the binding ability between IGF2BP3 and natural compounds, including triptolide (A), medicarpin (B), curcumin (C), curbitacin B (D), and epigallocatechin (E). Figure S2. The auto‐docking, molecular dynamics (MD), and proteomic analysis of CEL and IGF2BP3. The 3D (A) and 2D (B) binding conformations between CEL and IGF2BP3 (6GQE). The RMSD curve of the MD process of the CEL/IGF2BP3 complexes (PDB ID: 6GQE) (C: proteins; D: CEL). (E) The radius of gyration curves of the MD process of docked complexes (CEL/IGF2BP3(PDB ID: 6GQE). The comparison of conformations before and after MD simulations of the CEL/IGF2BP3 complexes (PDB ID: 6GQE) (F: 0 ns; G: 100 ns). The RMSD curve of the MD process of the CEL/IGF2BP3 complexes (PDB ID: 6FQR) (H: proteins; I: CEL). (J) The radius of gyration curves of the MD process of the docked complexes CEL/IGF2BP3 (PDB ID: 6FQR). The comparison of conformation before and after MD simulations of the CEL/IGF2BP3 complexes (PDB ID: 6FQR) (K: 0 ns; L: 100 ns). (M) Mass spectrum peak of the IGF2BP3 protein. Figure S3. CEL inhibits RA‐FLS proliferation and M1 macrophage polarization. (A) The viability of RA‐FLS was analyzed by the CCK8 assay. (B) The protein expression levels of IGF2BP3 in RA‐FLS after treatment with CEL for 24 h. Representative images of scratch assays (C) and Transwell assays (D) of RA‐FLS treated with CEL for 24 h. (E) The effect of CEL on F‐actin expression in RA‐FLS. (F) TUNEL (green) staining of RA‐FLS. (G) RA‐FLS apoptosis is measured with an annexin V‐FITC/PI staining assay after CEL treatment for 24 h. (H) Flow cytometric analysis is used to evaluate the cell cycle distribution of RA‐FLS. (I) The CCK8 assay is used to examine the viability of RAW264.7 after CEL treatment for 24 h. (J) The protein expression levels of IGF2BP3 in RAW264.7 cells after CEL treatment for 24 h. The proportion of CD86+ cells (K) and ROS content (L) in RAW264.7 cells treated with control, LPS, or CEL. *p < 0.05, [file MCO2-6-e70431-s001.pdf]

A new m^{6}A inhibitor, celastrol, alleviates rheumatoid arthritis via targeting IGF2BP3

Qishun Geng[1, 2], Yi Jiao[3, 5], Wenya Diao[3, 5], Jiahe Xu[4], Zhaoran Wang[2, 3], Xing Wang[3, 4], Zihan Wang[5, 6], Lu Zhao[7], Lei Yang[8], Yilin Wang[10], Kan Wang[11*], Tingting Deng[3*], Bailiang Wang[9*], Cheng Xiao[2, 3,12*]

[1] Department of Pharmacy, The First Affiliated Hospital of Zhengzhou University, Zhengzhou, 450052, Henan, China
[2] China-Japan Friendship Clinical Medical College, Chinese Academy of Medical Sciences & Peking Union Medical College, Beijing, 100029, China
[3] Institute of Clinical Medical Sciences, China-Japan Friendship Hospital, Beijing, 100029, China
[4] Peking University China-Japan Friendship School of Clinical Medicine, Beijing, 100029, China.
[5] Beijing University of Chinese Medicine, China-Japan Friendship Hospital Clinical Medicine, Beijing, 100029, China
[6] Department of TCM Rheumatology, China-Japan Friendship Hospital, Beijing, 100029, China
[7] China-Japan Friendship Hospital, Capital Medical University, Beijing, 100029, China
[8] Department of Pathology, China-Japan Friendship Hospital, Beijing, 100029, China
[9] Department of Orthopaedic Surgery, China-Japan Friendship Hospital, Beijing, 100029, China
[10] Beijing Friendship Hospital, Capital Medical University, Beijing, 100050, China
[11] Department of Anesthesiology, China-Japan Friendship Hospital, Beijing, 100029, China
[12] Department of Emergency, China-Japan Friendship Hospital, Beijing, 100029, China
*Correspondence: Kan Wang, wangkan2002@126.com
Tingting Deng, ttdeng1983@163.com
Bailiang Wang, orthopaedic_wang@126.com
Cheng Xiao, xc2002812@126.com

Supplementary material

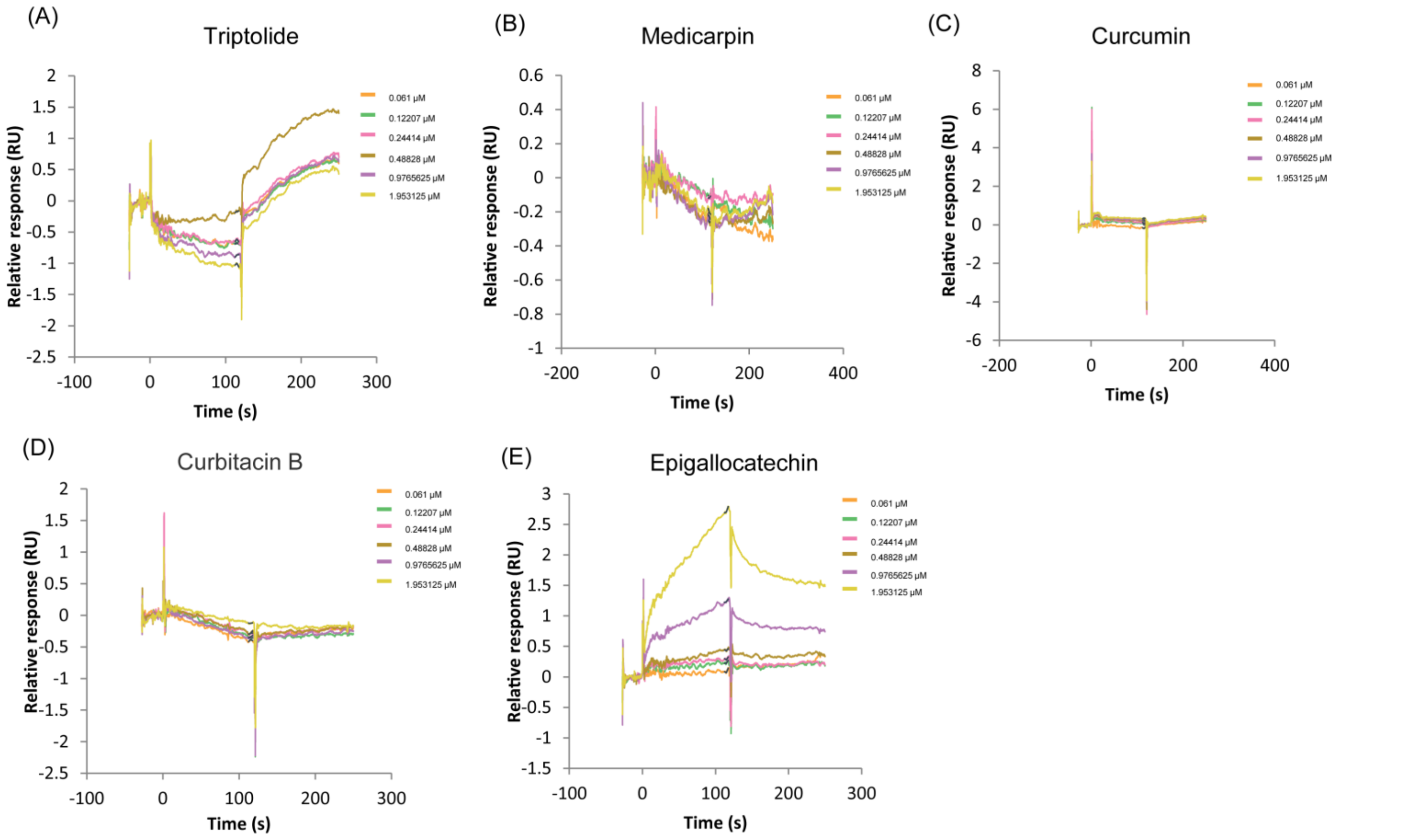

Figure S1 SPR was used to determine the binding ability between IGF2BP3 and natural compounds, including of Triptolide (A), Medicarpin (B), Curcumin (C), Curbitacin B (D), and Epigallocatechin (E).

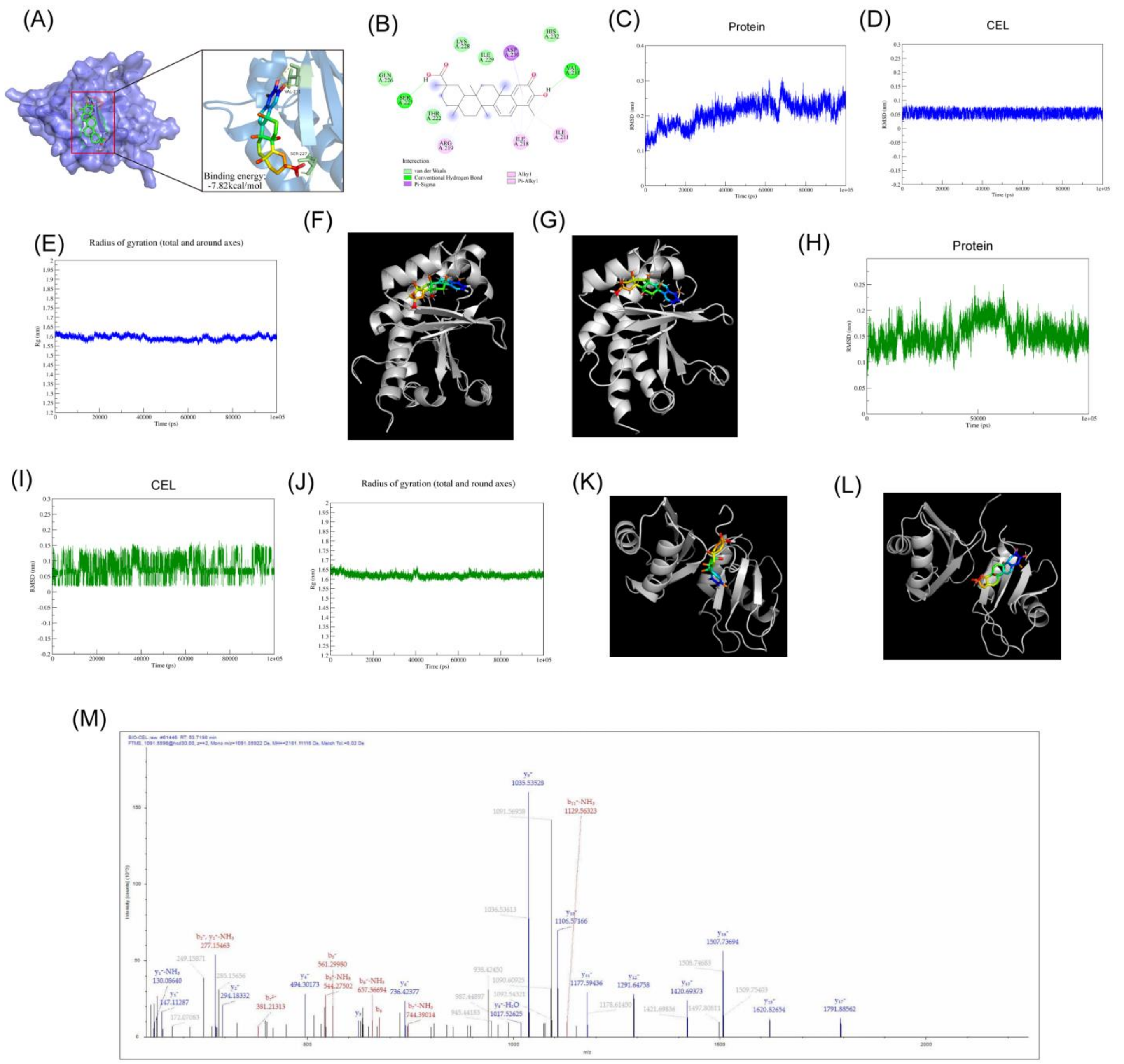

Figure S2 The autodocking, molecular dynamics and proteomic analysis between CEL and IGF2BP3. The 3D (A) and 2D (B) binding conformations between CEL and IGF2BP3 (6GQE). The molecular dynamics process' RMSD curve of the CEL/IGF2BP3 complexes (PDB ID: 6 GQE) (C: proteins, D: CEL). (E) The radius of gyration curves of the molecular dynamics process of docked complexes (CEL/IGF2BP3(PDB ID: 6GQE). The comparison of conformations before and after molecular dynamics simulations of the CEL/IGF2BP3 complexes (PDB ID: 6 GQE) (F: 0 ns, G: 100 ns). The molecular dynamics process' RMSD curve of the CEL/IGF2BP3 complexes (PDB ID: 6 FQR) (H: proteins, I: CEL). (J) The radius of gyration curves of the molecular dynamics process of the docked complexes (CEL/IGF2BP3(PDB ID: 6FQR). The comparison of conformation before and after molecular dynamics simulations of the CEL/IGF2BP3 complexes (PDB ID: 6FQR) (K: 0 ns, L: 100 ns). (M) Mass spectrum peak of the IGF2BP3 protein.

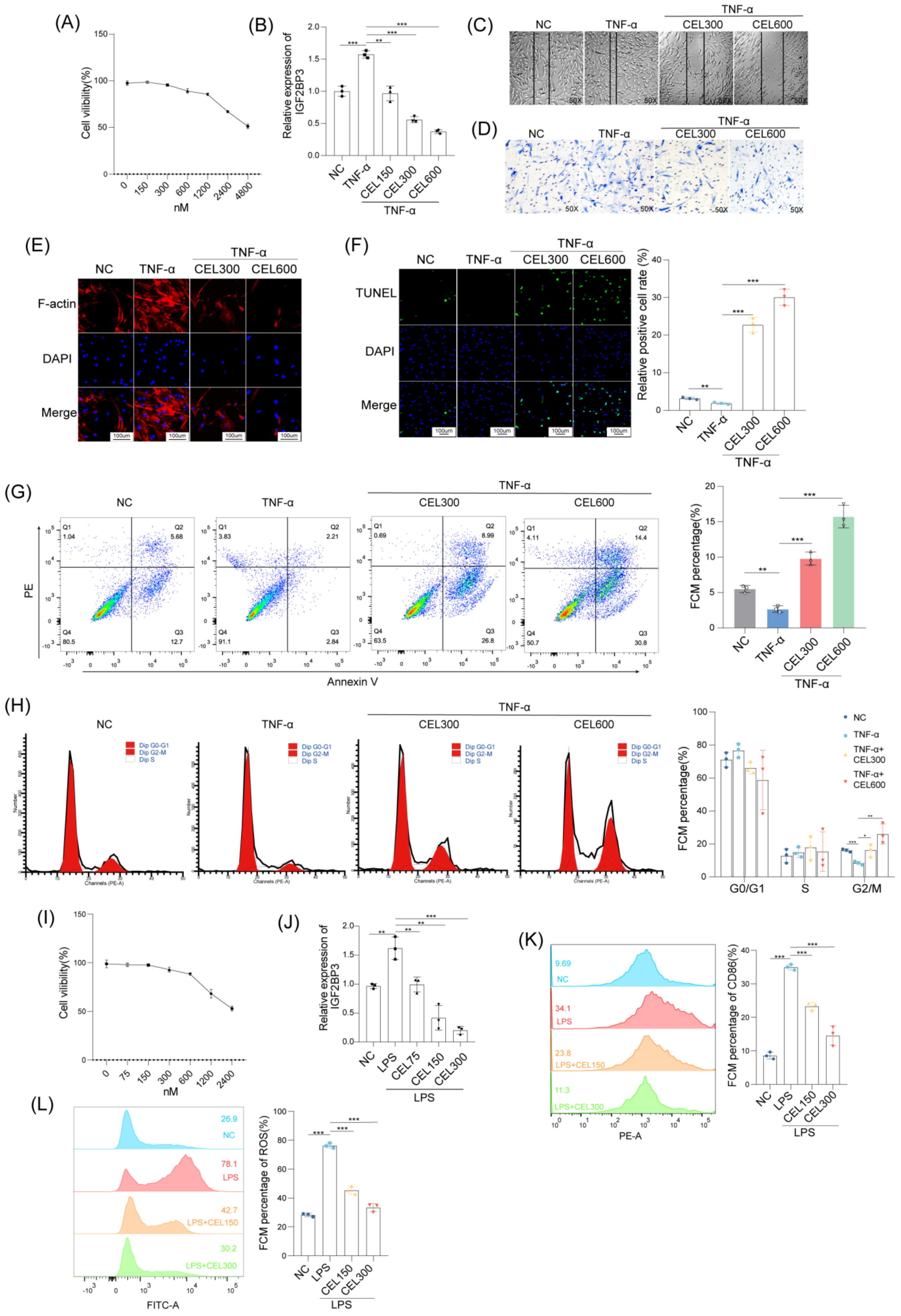

Figure S3 CEL inhibits RA-FLS proliferation and M1 macrophage polarization. (A) The viability of RA-

FLS was analyzed by the CCK8 assay. (B) The protein expression levels of IGF2BP3 in RA-FLS after treatment with CEL for 24 h. Representative images of scratch assays (C) and transwell assays (D) of RA-FLS treated with CEL for 24h. (E) The effect of CEL on F-actin expression in RA-FLS. (F) TUNEL (green) staining of RA-FLS. (G) RA-FLS apoptosis was measured with an annexin V-FITC/PI staining assay after CEL treatment for 24 h. (H) Flow cytometric analysis was used to evaluate the cell cycle distribution of RA-FLS. (I) The CCK8 assay was used to examine the viability of RAW264.7 after CEL treatment for 24 h. (J) The protein expression levels of IGF2BP3 in RAW264.7cells after CEL treatment for 24 h. The proportion of $CD86^+$ cells (K) and ROS content (L) in RAW264.7 cells treated with control, LPS or CEL. *P < 0.05, **P < 0.01, ***P < 0.001.

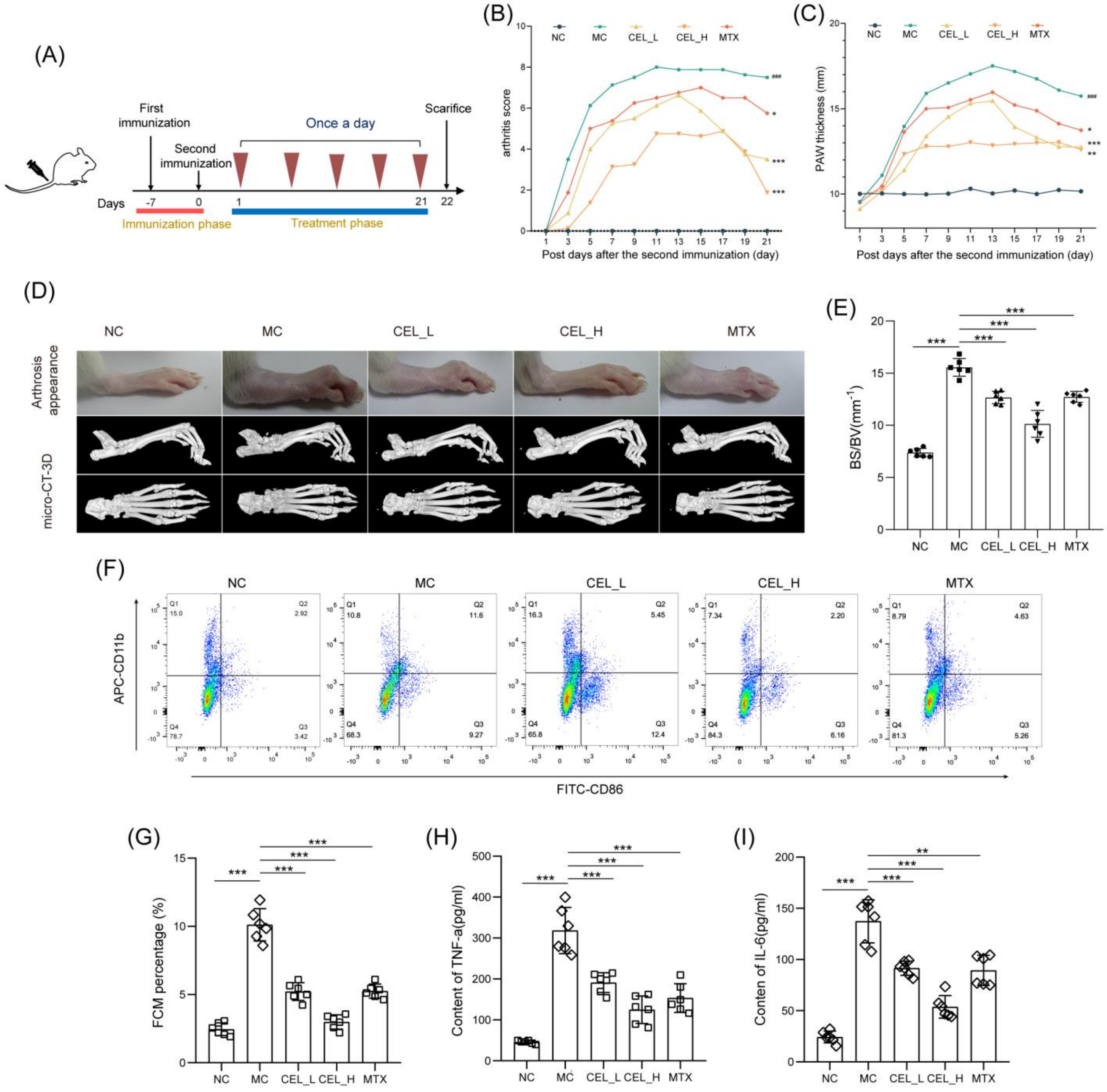

Figure S4 CEL alleviates arthritis progression in CIA rats. (A) An illustration of the induction and treatment of rats with CIA. (B) Arthritis scores were monitored once every 3 days. (C) Hind paw thickness was calculated after the second immunization. Compared with the NC group, #p<0.05, ##p<0.01, ###p<0.001. Compared with the MC group, *p<0.05, **p<0.01, ***p<0.001. (D) Paw photographs of rats on 22 day and the micro-CT analysis of paws. (E) BS/BV in the ankle was examined by micro-CT. (F-G) The proportions

of CD45⁺CD11b⁺CD86⁺ cells in the spleens of rats. The contents of TNF-α (H) and IL-6 (I) in the serum of rats. *p<0.05, **p<0.01, ***p<0.001.

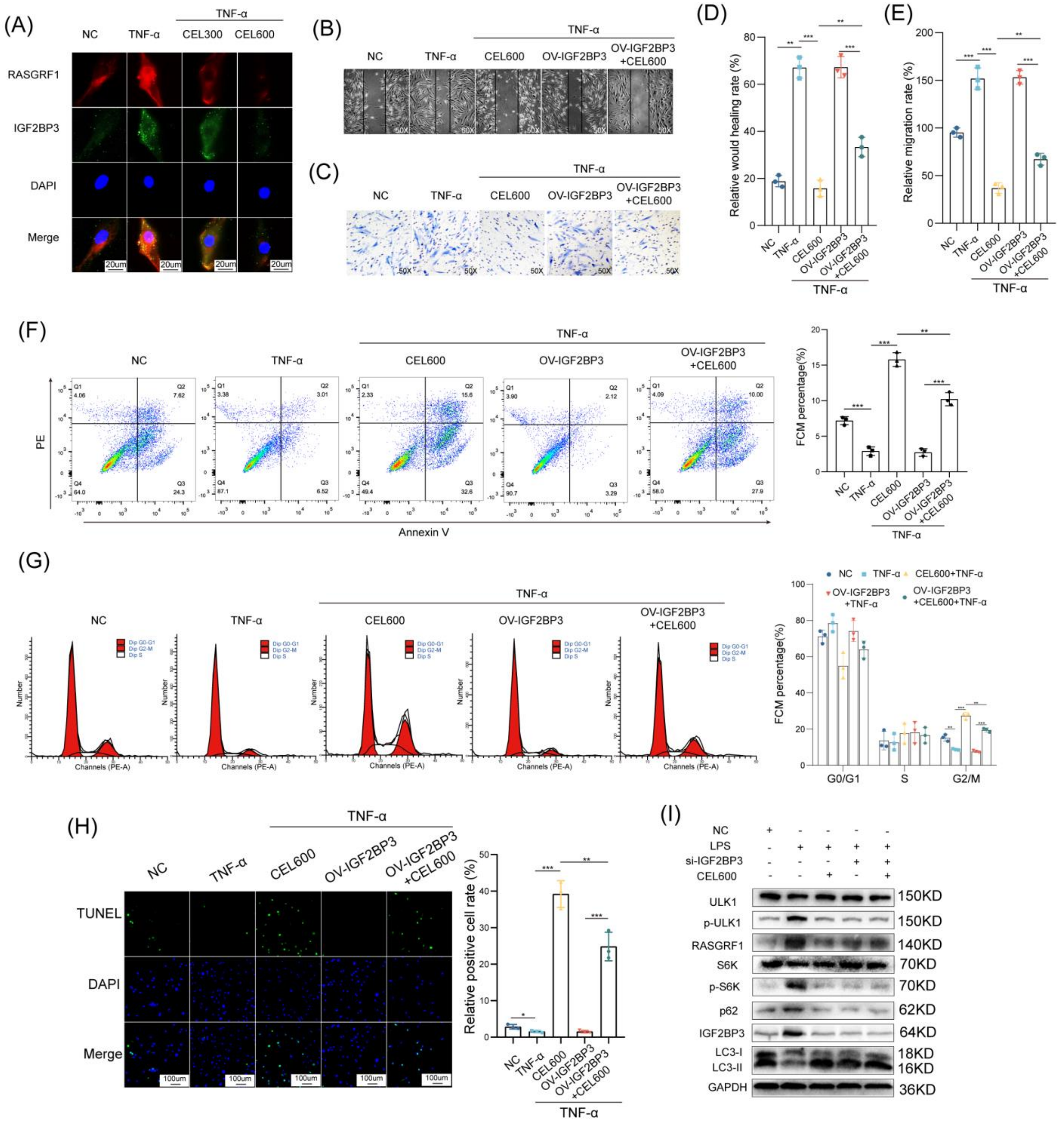

Figure S5 IGF2BP3 is a direct target of CEL to inhibit cell proliferation and inflammatory activation. (A) The immunofluorescence staining of IGF2BP3 and RASGRF1 in RA-FLS after treatment with TNF-α or CEL. The quantification of scratch healing assay (B, D) and transwell assay (C, E) in RA-FLS after treatment with TNF-α, CEL or IGF2BP3 overexpression. (F) The percentage of apoptotic RA-FLS was measured with an annexin V-FITC/PI staining assay. (G) Flow cytometric analysis was used to evaluate the cell cycle distribution of RA-FLS. (H) Tunnel (green) staining in RA-FLS. (I) Western blot analysis of the levels of ULK1, p-ULK1, S6K, p-S6K, RASGRF1, IGF2BP3, p62 and LC3 in RA-FLS after treatment with TNF-α, CEL or siIGF2BP3. *p < 0.05, **p < 0.01, ***p < 0.001.

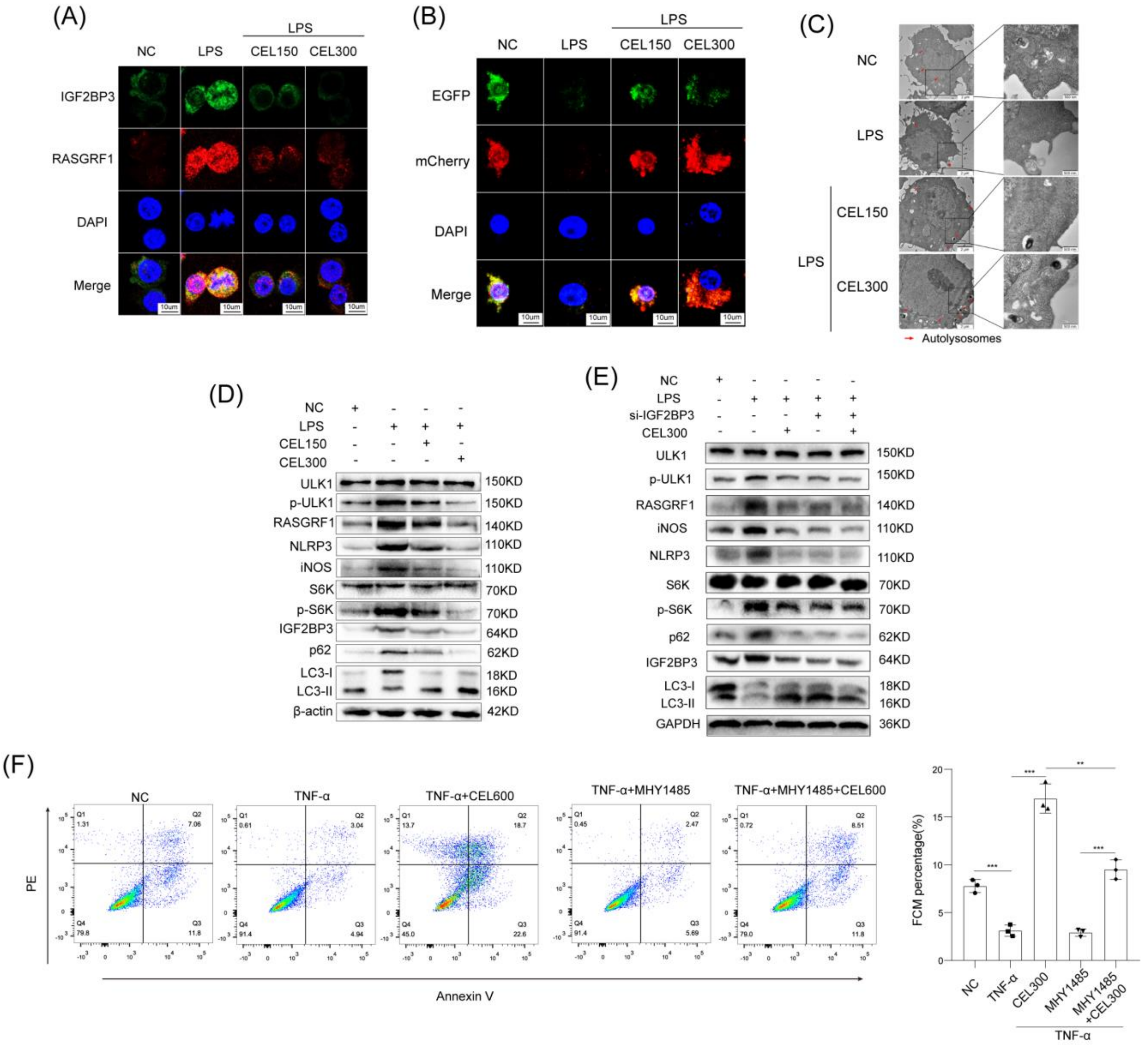

Figure S6 IGF2BP3-mediated mTORC1 activation plays a significant role in CEL-mediated inhibition of cell proliferation and inflammatory activation. (A) The immunofluorescence staining of IGF2BP3 and RASGRF1 in RAW264.7 cells treated with CEL or LPS for 24 h. (B) Representative images of RAW264.7 cells expressing mCherry-GFP-LC3. (C) TEM analysis of RAW264.7 cells treated with LPS or CEL. (D) Western blot analysis of ULK1, p-ULK1, S6K, p-S6K, NLRP3, iNOS, RASGRF1, IGF2BP3, p62 and LC3 in THP-1 cells after treatment of LPS or CEL. (E) Western blot analysis of ULK1, p-ULK1, S6K, p-S6K, iNOS, NLRP3, RASGRF1, IGF2BP3, p62 and LC3 in RAW264.7 cells after treatment with LPS, CEL or siIGF2BP3. (F) Flow cytometric analysis was used to evaluate the apoptosis proportion of RA-FLS treated with control, LPS, CEL or MHY1485. *$p < 0.05$, **$p < 0.01$, ***$p < 0.001$.

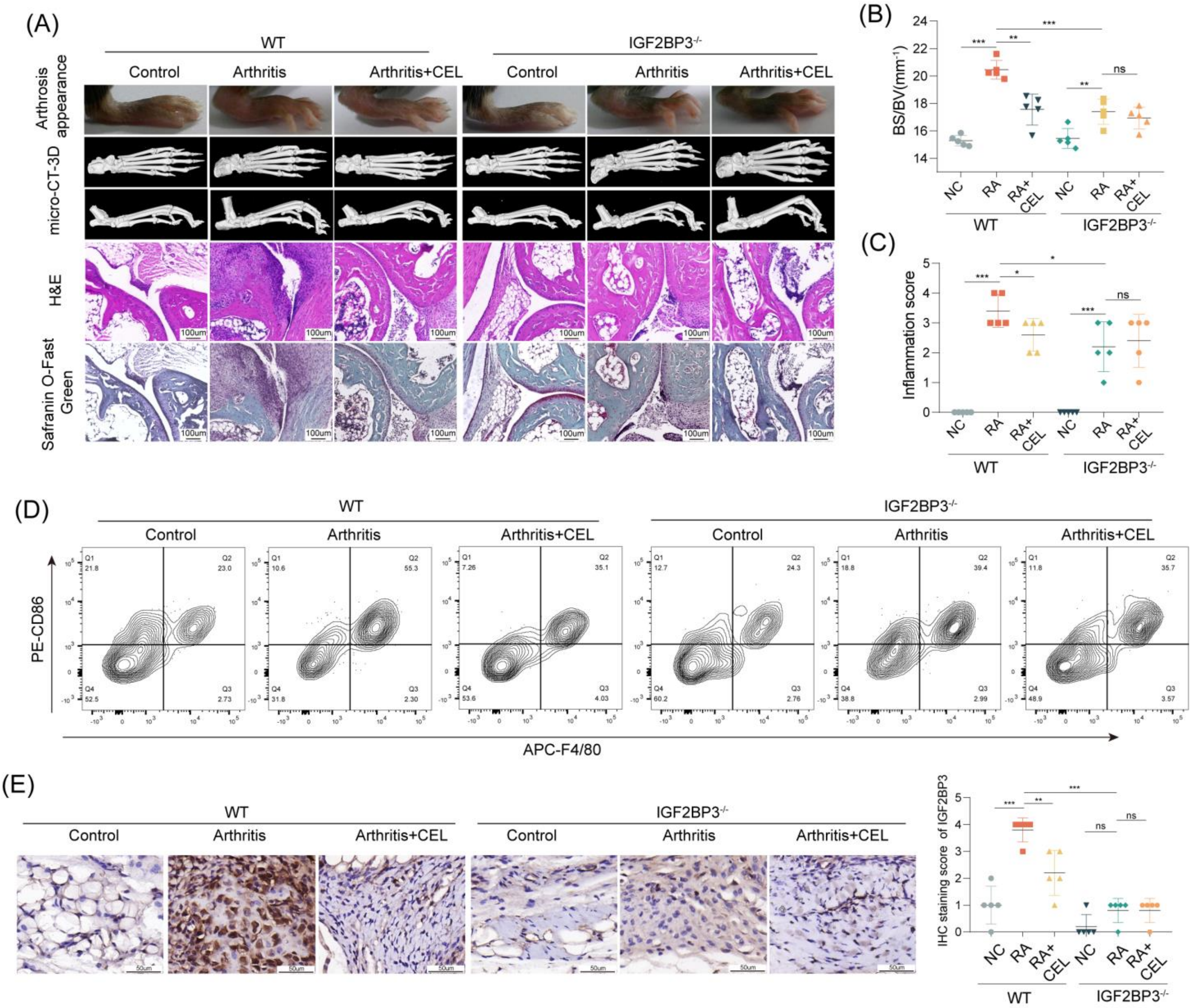

Figure S7 The knockout of IGF2BP3 abolishes the effects of CEL in alleviating the progression of RA. (A) Representative histology images of H&E, Safranin O/Fast green staining, Arthrosis appearance and micro-CT were obtained from mice' ankle. (B) BS/BV in the ankle was measured by micro-CT. (C) The H&E scores were assessed. (D) The proportion of F4/80^{+}CD11b^{+}CD86^{+} M1 macrophages in spleens of mice. (E) Representative immunohistochemical assays and scores of IGF2BP3 in mice' synovial tissue. *p < 0.05, **p < 0.01, ***p < 0.001.

Table S1 The sequences of the primers used for RT_qPCR.

**Gene**	**Forward sequence (5′-3′)**	**Reverse sequence (5′-3′)**
Human		
TNF-α	GAGGCCAAGCCCTGGTATG	CGGGCCGATTGATCTCAGC
IL-17	TCCCACGAAATCCAGGATGC	GGATGTTCAGGTTGACCATCAC
MMP3	AGGCAAGACAGCAAGGCATA	ACGCACAGCAACAGTAGGAT
RASGRF1	TCCTGTCGTGAACTGGACAAT	CTGCACTGGCTAAGGACATCC
IGF2BP3	ACGAAATATCCCGCCTCATTTAC	GCAGTTTCCGAGTCAGTGTTCA
β-actin	CCTTCCTGGGCATGGAGTC	TGATCTTCATTGTGCTGGGTG
Mouse		
RASGRF1	GCCAGAAGACTTGACAACGCT	TCAATCTACAGGGATGGTGGAAG
IGF2BP3	CCTGGTGAAGACGGGCTAC	TCAACTTCCATCGGTTTCCCA
NLRP3	ATTACCCGCCCGAGAAAGG	TCGCAGCAAAGATCCACACAG
iNOS	GTTCTCAGCCCAACAATACAAGA	GTGGACGGGTCGATGTCAC
Gapdh	AAATGGTGAAGGTCGGTGTGAAC	CAACAATCTCCACTTTGCCACTG
